# Carbon isotope and soluble metabolites reflect physiological status among contrasting faba bean genotypes in response to water deficit

**DOI:** 10.3389/fpls.2022.955406

**Published:** 2022-09-16

**Authors:** Md Abdul Muktadir, Andrew Merchant, Abdus Sadeque, Mohsin Tanveer, Kedar Nath Adhikari, Liping Huang

**Affiliations:** ^1^Faculty of Science, School of Life and Environmental Sciences, The University of Sydney, Sydney, NSW, Australia; ^2^International Research Center for Environmental Membrane Biology, College of Food Science and Engineering, Foshan University, Foshan, China; ^3^Faculty of Science, Plant Breeding Institute, The University of Sydney, Sydney, NSW, Australia; ^4^Pulses Research Centre, Bangladesh Agricultural Research Institute, Gazipur, Bangladesh; ^5^Tasmanian Institute of Agriculture, University of Tasmania, Hobart, TAS, Australia

**Keywords:** carbon isotope, grain legumes, physiology, sugars, WUE

## Abstract

Identification and validation of biomarkers and bioindicators to select genotypes with superior tolerance to water deficit (WD) under field conditions are paramount to plant breeding programs. However, the co-occurrence of different abiotic stresses such as WD, heat, and radiation makes it difficult to develop generalized protocols to monitor the physiological health of the plant system. The study assessed the most abundant carbohydrates and sugar alcohols in five faba bean (*Vicia faba*) genotypes under field conditions and the abundance of naturally occurring carbon isotopes in bulk leaf material to predict water use efficiency (WUE). Plant water status and biomass accumulation were also assessed. Among the accumulated sugars, inter-specific variation in glucose was most prevalent and was found at a higher concentration (8.52 mg g^−1^ leaf) in rainfed trial. *myo-*Inositol concentrations followed that of glucose accumulation in that the rainfed trial had higher amounts compared to the irrigated trial. WUE calculated from carbon isotope abundance was consistently offset with measured WUE from measurements of leaf gas exchange. All genotypes demonstrated significant relationships between predicted and measured WUE (*p* < 0.05) apart from control variety PBA Warda. Thus, bulk leaf-level carbon isotope abundance can be used to calculate WUE and used as an effective selection criterion for improving WUE in faba bean breeding programs under field conditions.

## Introduction

Among abiotic stresses, water deficit (WD) is the most influential on faba bean yield and production (Daryanto et al., 2015). Systematic research on WD can advance yield improvement for water-limited cultivation (Hsiao et al., 2007). Novel traits that indicate plant water status can inform selection programs to identify superior lines for both high yield and increased resilience of yield (Smith et al., 2019). In water-limited environments, water use efficiency (WUE) is found to be one of the most important traits for yield improvement across a range of legume crops (Blessing et al., 2018). Plants deploy a range of approaches to cope with WD, including enhanced efficiency of use through reduced stomatal conductance (g_s_) and enhanced metabolites acquisition to maintain tissue hydration (Iannucci et al., 2002; Serraj and Sinclair, 2002). Genotypes that can assimilate carbon at low g_s_ are often considered to be tolerant to WD based on the assumption that sustained growth will equally maintain yield. Thus, WUE is therefore the focus of targeted screening tools for many crops (Loss et al., 1997; Xu and Hsiao, 2004; Kashiwagi et al., 2013) to identify the genotypes that can sustain yield during periods of WD. At the leaf level, WUE can be calculated by dividing net photosynthesis (A) by g_s_, termed instantaneous WUE (Chaves and Oliveira, 2004), or can be estimated *via* isotopic fractionation models, termed intrinsic WUE (Farquhar et al., 1982). Generally, instantaneous WUE involves measurement of gas exchange at a single point while periodic measurement throughout the day is also suggested (Cernusak et al., 2005; Smith et al., 2016). Periodic measurements often can be beyond the capabilities of researchers to study large populations. Intrinsic WUE therefore offers advantages in both cost and time integration (Merah et al., 2001; Teulat et al., 2001; Monneveux et al., 2004). However, few of these studies consider the embedded differences in components of the fractionation model with that of time-course integrated quantification of instantaneous measures of WUE.

Accumulation of chemical entities due to stresses (often termed “stress metabolites”) may give an indication of plant responses against specific stress (Weckwerth, 2011). However, most “metabolomic” studies conducted under control conditions involve water, heat, and light stress either singularly or in a combination to identify/quantify metabolites accumulating under these stresses. Among the major metabolic groups, carbohydrates and sugar alcohols play a vital role to alleviate adverse effects of stress through osmolytic and osmo-protective mechanisms (Loescher, 1987; Bohnert and Shen, 1998; Merchant and Richter, 2011). To date, no study has identified the accumulation of stress metabolites in faba bean.

Despite a relative paucity of chemical and physiologically based pre-breeding strategies, global yields of faba bean are increasing with a corresponding reduction in harvested area over the last 50 years (Foyer et al., 2016). At the crop scale, unstable yield production, especially due to abiotic factors, makes faba bean a “risky” crop among cultivated grain legumes. Genotypic differences in WD responses exist, but the underlying mechanisms are yet to be understood (Link et al., 1999; Khazaei et al., 2013). Genetic advancement for this crop requires the identification of physiological and biochemical responses due to WD under field conditions for use in plant improvement programs.

Faba bean genotypes were selected based on the contrasting carbon isotope discrimination (Δ) values in an earlier field trial (Muktadir, 2019). This study subsequently investigated the suitability of Δ isolated from leaves as a surrogate for plant gas exchange measurement in five diverse faba bean genotypes under irrigated and rainfed conditions. Quantification of metabolites is also determined to investigate the relative changes in concentration under WD conditions. WUE from field-based gas exchange was compared to that calculated from leaf-level Δ to assess the presence of any systematic differences influencing the ranking of individuals in a breeding program. These parameters are presented on a background of biomass accumulation and yield under rainfed and irrigated conditions.

The hypotheses were therefore (i) rainfed conditions will induce a reduction in stomatal conductance across all faba bean genotypes with some variation and the reduction will be lower in tolerant genotypes; (ii) differences in water availability will elicit qualitative and quantitative changes in the concentration of soluble metabolites in leaves; (iii) WUE calculated from gas exchange (instantaneous) and calculated from leaf-level Δ (intrinsic) will be influenced by water availability, indicating continued carbon uptake during stress; and (iv) intrinsic WUE calculated from isotope fractionation will be systematically offset with instantaneous WUE integrated across the daily time course—hence function as a surrogate measure of WUE for plant breeding programs.

## Methodology

### Plant materials and experimental design

Based on isotope discrimination, four contrasting faba bean genotypes were selected (AC0805#4912, 11NF020a-1, 11NF010c-4, and 11NF008b-15) along with PBA Warda, a popular variety suitable for the grain-growing region of northern NSW, Australia. The experiment was carried out at the I.A. Watson Grains Research Centre, Narrabri, NSW-2390, Australia (30° 27′ S, 149° 80′ E), in 2016 (7 May to 29 October). The initial screening experiment was conducted at the same location in 2014 (Muktadir, 2019). Each genotype was sown in four replicates of individual plots comprising four rows 10-m long and 50-cm apart. Two treatments were maintained irrigated and rainfed. Irrigated and rainfed blocks were separated by a buffer plot of PBA Warda variety (48 × 12 m).

### Experimental field management

**Figure 1:**
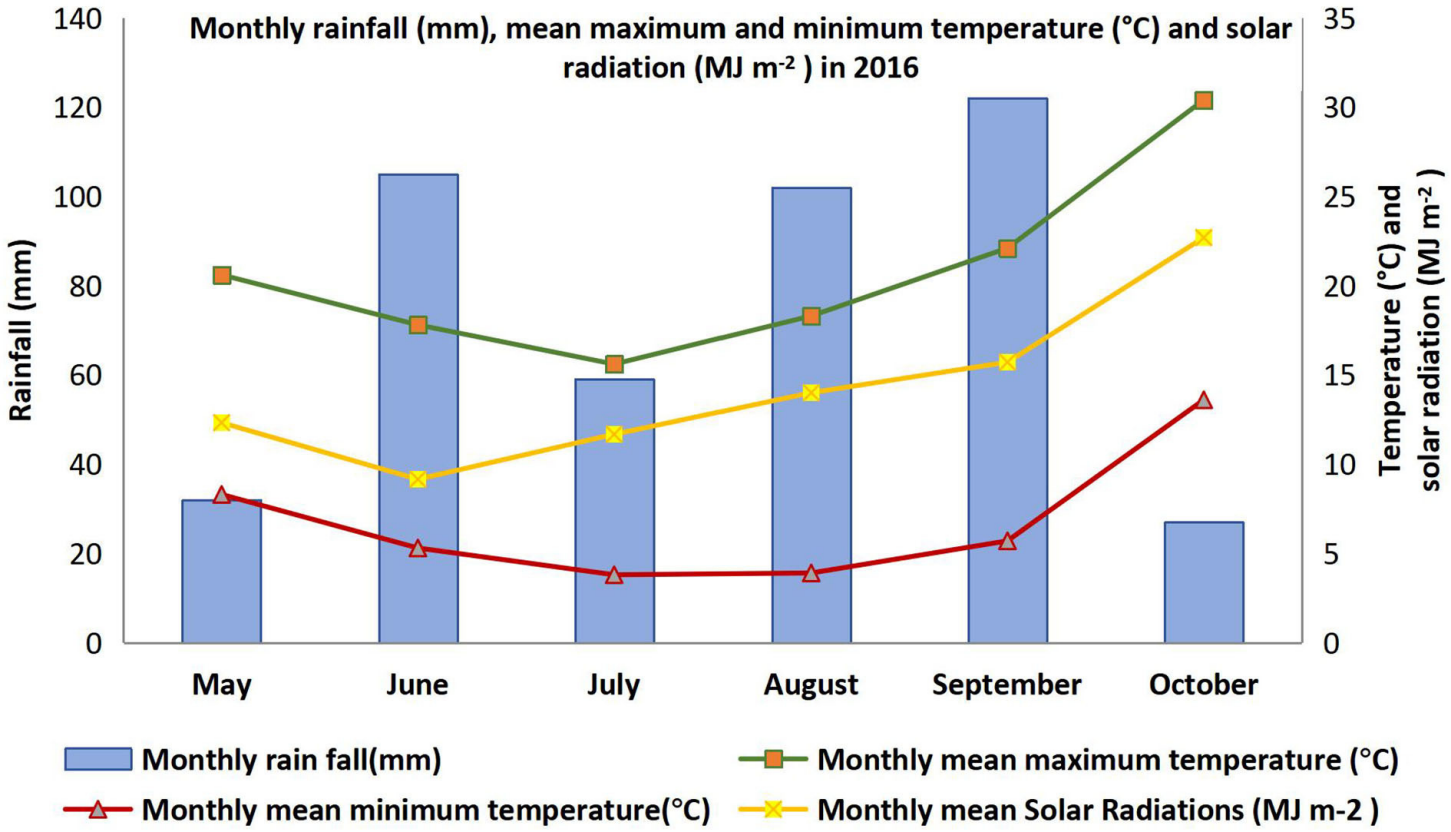
Monthly rainfall (mm), mean maximum and minimum temperature (°C), and solar radiation (MJ m^−2^) in 2016 at Narrabri.

The soil at the experimental site was vertosol (Isbell, 1996) with pH 8.2. The experiment was sown on 7 May 2016 in a field that had wheat in the previous crop season, plowed and leveled. Seeds were pretreated with commercially available faba bean inoculant (*Rhizobium* strain, group G WSM 1455). Pre-emergence and post-seeding weedicides, namely, Spinnaker 700WG (Imazethapyr 700 g kg^−1^) and Terbyne 750 WG (teerbuthylazine 750 g kg^−1^), were applied at a recommended rate to prevent the establishment of a range of grass and broadleaf weeds. Karate Zeon® (250 g L^−1^ lambda-cyhalothrin) at the rate of 36 ml ha^−1^ was applied to control insect pests as regular farm practices during 50% flowering. Mancozeb (750 g kg^−1^) was sprayed before the canopy closure (1.7 kg ha^−1^) to control rust (*Uromyces viciae-fabae*) and chocolate spot (*Botrytis fabae*). No fertilizer was applied during crop growth. The season was rainfall-dominated and rainfed plots also received 447 mm rainfall over the season. The irrigated plots received a total of 472 mm during this period, which is only 25 mm more than the rainfed plot. Although both conditions received almost the same amount of moisture over the season, the distribution was uneven in rainfed plots, whereas irrigated plots received irrigation on a regular basis. Daily maximum and minimum temperature, and monthly mean solar radiation, along with accumulated rainfall data during crop season, were recorded through a data logger placed near the field trial. The photoperiod was 11 h and 30 min on the measurement days. The detail of weather data for the growing period (May to October 2016) is presented in Figure 1. Grain yield was weighed after harvesting trial plots by using a mechanical harvester (HALDRUP C-65).

### Gas exchange measurements

Leaf-level photosynthesis was measured from 09:00 to 16:00 on the same leaflet at least four times over the day on four randomly selected plants from each plot using a Walz GFS-3000 portable gas exchange system. Leaf temperature and chamber CO_2_ concentration, along with air humidity, was set at ambient. Data were completed on two consecutive days where day 1 was allocated for irrigated and day 2 for rainfed trial. The average temperature was 24.2 and 21.3°C, average relative humidity was 60.2 and 62.3%, and average PAR was 1.543 and 1.523 μmol m^−2^ s^−1^ on sampling day 1 and sampling day 2, respectively. Light source during gas exchange measurements was set at tracking mode, i.e., natural light source. After insertion of leaves into the gas exchange chamber, leaves were allowed to stabilize with chamber conditions until steady-state gas exchange rates were established. Leaves were chosen from the main stem (second/third fully expanded).

### Leaf water content and biomass measurement

The adjacent leaf that was tagged for gas exchange measurements was used to get leaf water potential. The leaflets were excised and transported (within 15 min) in a zip-locked bag over ice to preserve water content for subsequent measurement with a Wescor PSYPRO. Samples for water relations were collected immediately before sunrise at ~6:30 during sampling days. Relative water content (RWC) was also determined from the adjacent leaf, which was used to measure leaf water potential. Leaves were placed in distilled water and allowed to soak overnight at 4°C. Subsequently, leaves were weighed immediately to have a turgid weight. Finally, leaves were dried in an oven at 65°C for 48 h to have a constant weight. RWC was determined by


RWC (%) = [(FW−DW) / (TW−DW)] × 100

(*Barrs and Weatherley*, 1962)

where FW is the fresh weight of leaves, TW is the turgid weight of leaves, and DW is the oven dry weight of leaves (Barrs and Weatherley, 1962).

At physiological maturity, 1 m^2^ of each plot was harvested and biomass placed in a polyethylene mesh bag until dried (sun-dried) to determine biological yield. After weighing the plants, samples were threshed to determine grain yield. Finally, harvest index (HI) was measured according to the formula


$$\begin{array}{l} \text{HI }(\%)\;=\;\text{Grain yield}/\text{Biological yield}\times 100. \end{array}$$

Harvesting of the whole plot was completed by mechanical harvester upon maturity and plot yield converted into kg ha^−1^.

### Leaf tissue collection and carbon isotope analysis

Leaf samples for chemical and isotopic analysis were collected on the same day immediately after gas exchange measurement between 16:00 and 17:00. Measures of carbon isotope abundance were completed on the same tagged leaf, which was used for the measurement of gas exchange. Leaves were cut with a razor and immediately put into 2-ml Eppendorf tube and placed in an ice box to reduce metabolism. Within 2 h, samples were put in an oven (65°C for 48 h) to completely dry. An oscillating matrix mill was used to grind the dried leaf sample. Then, 3 mg of ground leaf was poured into silver capsules (IVA Analysentechnik, Meerbusch, Germany) and then analyzed according to the protocol outlined in Smith et al. (2019).

### Quantification of metabolites

Dried leaf samples were transferred to 2-ml microcentrifuge tubes and grounded using an oscillating matrix mill. Methanol–chloroform–water (MCS) extractions were then performed according to Merchant et al. (2006). Approximately 40 mg of ground leaf samples was weighed into 2-ml screw cap tubes, and the exact weight for each sample was recorded. For the MCW solution (12:5:3 by volume), the water fraction of the extraction solution contained 0.1% pentaerythritol (98+%, Alfa Aesar, Haverhill, MA, USA) as an internal standard. Then, 1 ml of the solution was added to each sample and incubated at 70°C for 30 min, then allowed to cool for 5 min before centrifuging for 2 min at 10,000 × *g*. Then, 800 μl of supernatant was pipetted into clean 2-ml pop cap microcentrifuge tubes. Also, 500 μl of MilliQ water and 200 μl of chloroform were added to 800 μl of supernatant and mixed thoroughly with the vortex. The samples were then centrifuged for 2 min at 10,000 × *g* and then left to stand for 10 min. Then, 700 μl of the top aqueous phase was removed and added to a clean 1.7-ml microcentrifuge tube. The samples were then shaken at room temperature for 2 h and centrifuged for 2 min at 10,000 × *g*. Finally, 400 μl of supernatant was removed and pipetted into a clean, labeled 2-ml microcentrifuge tube and stored at −80°C until the samples could be run on the gas chromatography triple quadrupole mass spectrometer (GC-QQQ). In order to analyze nonpolar analytes using GC-QQQ, the samples had to be derivatized according to Merchant et al. (2006). The separation and quantification of target metabolites were completed using an Agilent 6890A gas chromatograph with QQQ 7000 mass selective detector on scan mode from 50 to 500 AMU (70 eV) (Agilent Technologies, Santa Clara CA, USA) according to the protocol detailed in Merchant et al. (2006). Metabolite concentrations are reported as mg g^−1^ dry weight sample material.

### Modeling of carbon isotope abundance

The predicted isotope values in plant components were calculated using the equation (Farquhar et al., 1989).


$$\begin{array}{l} \Delta =\text{ a}+\;(\text{b}-\text{a})\frac{\text{Ci}}{\text{Ca}}-\text{d}, \end{array}$$

where “a” is fractionation caused by gaseous diffusion through the stomata (4.4‰), “b” is the effective fractionation caused by carboxylating enzymes (approximately 27‰), “Ci” is the internal CO_2_ concentration (calculated by Walz GFS-300 software), “Ca” is the atmospheric CO_2_ concentration (set to ambient), and “d” is a generalized term combining fractionation during photorespiration, dark respiration, and dissolution and diffusion from the gaseous phase to the chloroplasts. For relationships with measured δ^13^C, assimilation weighted Ci was calculated (Cernusak et al., 2005) to account for proportional changes in the contribution of photosynthesis to δ^13^C across the course of the light period. This was achieved by the formula


$$\sum_{i=1}^{A}ci=\;\frac{A1}{\sum }Ci_{1+}\frac{A2}{\sum }Ci_{1+}\frac{An}{\sum }Ci_{\text{n}}$$

where A_1_ and Ci are the first observed A and Ci, respectively, in the light period. The Δ was then calculated as the difference between the isotopic composition of atmospheric CO_2_ (δ^13^C air ≈ −8‰) as carbon source and that of the plant organic matter (δ^13^C plant) as photosynthetic product using Δ (‰) = (δ^13^C air –δ^13^C plant)/1+ plant.

### Calculation of water use efficiency

Two calculations of WUE were completed in this study. Intrinsic WUE was calculated based upon gas exchange measures using the formula outlined by Osmond et al. (1980):


$$\begin{array}{l} \text{WUE}\;=\frac{\text{A}}{\text{gs}} \end{array}$$

where A is the net photosynthesis (μmol m^−2^ s^−1^ CO_2_), and g_s_ is the stomatal conductance (mmol m^−2^ s^−1^ H_2_O). Modeled WUE was calculated based upon isotope abundance from the formula outlined by Seibt et al. (2008), originally developed by Farquhar and Richards (1984):


$$\begin{array}{l} \text{WUE }=\;\frac{\text{Ca}}{1.6}\left(\frac{\text{b}-\Delta }{\text{b }-\text{ a}}\right) \end{array}$$

where the value “1.6” is the ratio of the diffusion rate between CO_2_ and H_2_O across the stomatal cavity, and “b” is the effective fractionation caused by carboxylating enzymes RuBisCO and PEP carboxylase (~27‰).

### Statistical analysis

The effects of treatments and genotype on leaf water content, gas exchange parameters, and carbohydrates concentration were examined by ANOVA using GenStat 18th Edition (VSN International, Hemel Hempstead, UK). Duncan's multiple-range tests were used to determine significant differences (*p* < 0.05) among genotypes. Regression analysis with best-fitting line and significance level (*p-*values) for the relationship of WUE was calculated using GraphPad Prism7 software (GraphPad Software, San Diego, CA, USA).

## Results

### Leaf water relations

**Figure 2:**
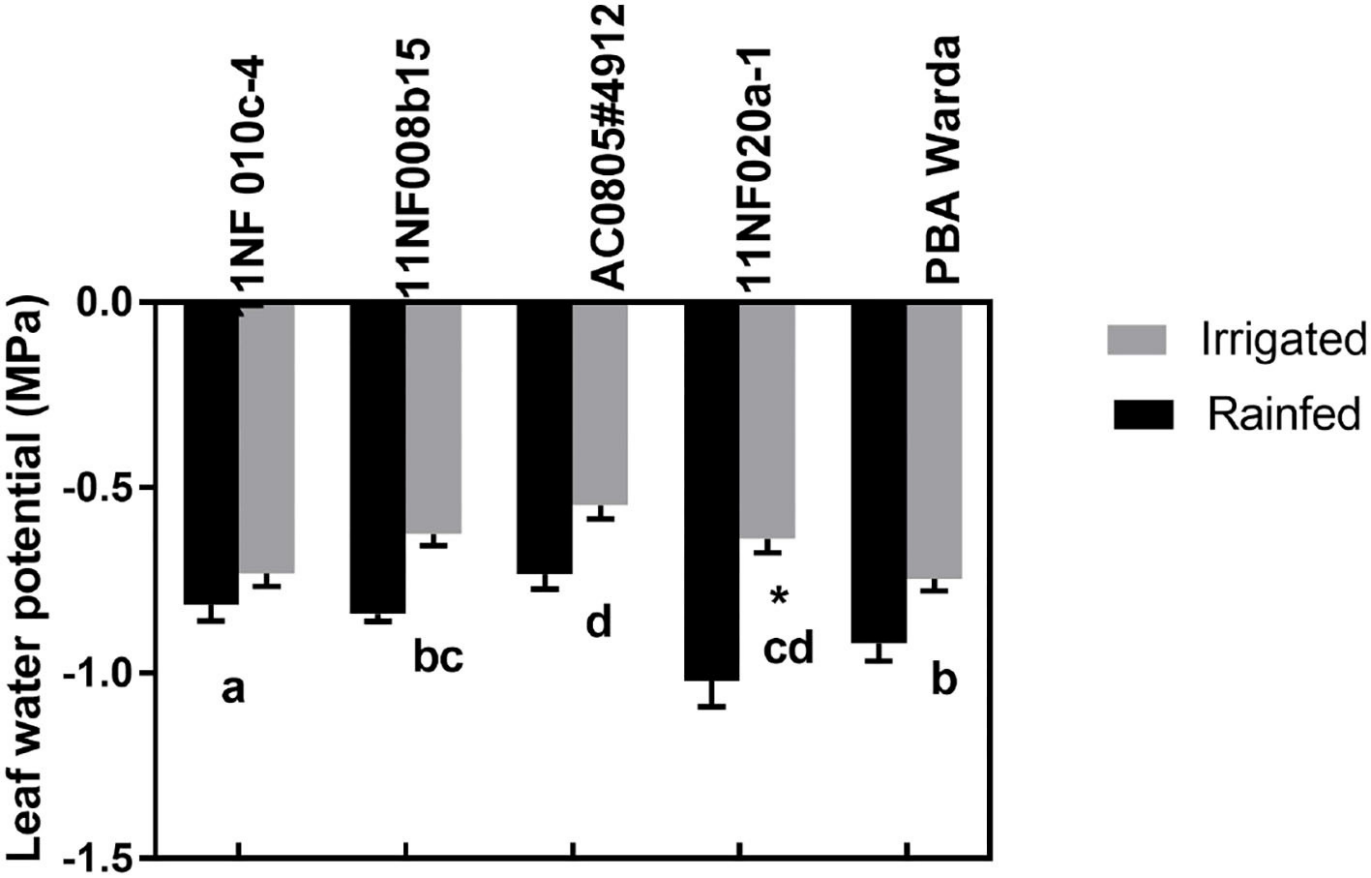
Predawn leaf water potentials (MPa) of contrasting faba bean genotypes at the three-pod stage grown under field condition (irrigated and rainfed trial) at Narrabri, Australia. Error bars were calculated from four replicates and represent standard error of the mean. Letters represents a significant difference (*p* < 0.05) among genotypes calculated through DMRT. Asterisks (*) represent significance between irrigated and rainfed treatments.

Leaf water potential was higher (less negative) in irrigated plots compared to rainfed plots for all genotypes at the pod-filling stage, although they were nonsignificant (Figure 2). Genotype had a significant effect. Among genotypes, 11NF010c-4 had the lowest water potential (most negative) and AC0805#4912 had the highest (least negative). No other genotypes except 11NF020a-1 differed according to treatment.

**Figure 3:**
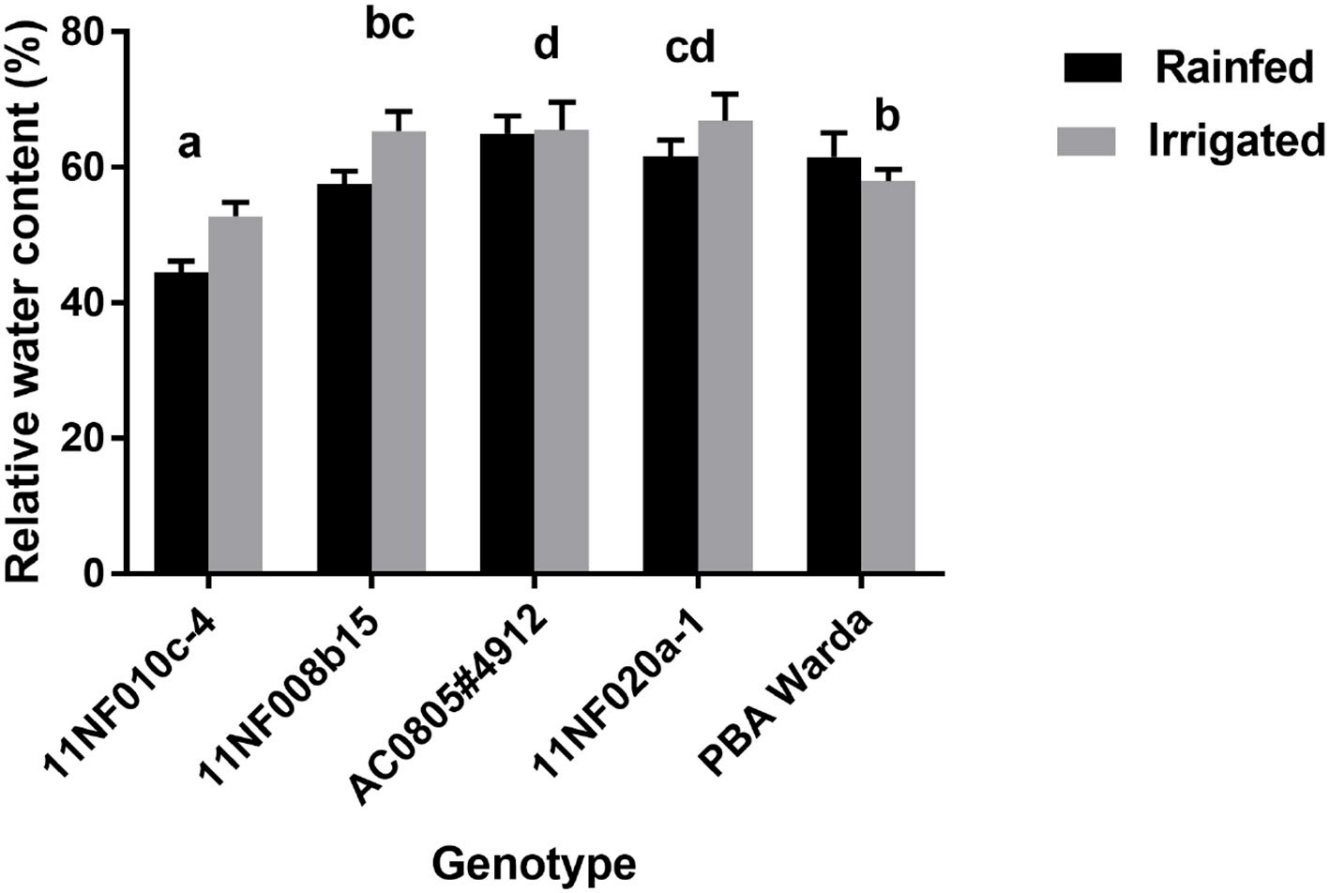
Relative water content (%) of contrasting faba bean genotypes grown under field conditions (irrigated and rainfed trial) at Narrabri, Australia. RWC was determined at the three-pod stage. Error bars were calculated from four replicates and represent standard error of the mean. Letters denote a significant difference (*p* < 0.05) among genotypes calculated through DMRT.

Relative water content was not significantly different between treatments. However, significant variation existed among genotypes. The highest RWC was observed in the genotype AC0805#4912, which was counted as a WD-tolerant genotype, and the lowest was in 11NF010c-4 (Figure 3).

### Leaf gas exchange

The mean transpiration rate in the irrigated condition was higher than the rainfed condition irrespective of genotypes. Among genotypes, AC0805#4912 had the highest for irrigated trial, whereas 11NF020a-1 had the highest for rainfed trial (Table 1). Photosynthetic rates also showed similar patterns to transpiration rate among genotypes and across the course of the day. Notably, genotype 11NF010c-4 showed a lower photosynthetic rate across the day for both irrigated and rainfed trials, whereas AC0805#4912 had the highest rate for both trials over the time period of data recording (Table 1).

**Table 1:** Mean transpiration rate (E, mmol m^−2^ ms^−1^), net photosynthetic rate (A, μmol m^−2^ ms^−1^), stomatal conductance (g_s_, mmol m^−2^ ms^−1^), and ratio between intercellular carbon concentrations (Ci) to atmospheric carbon concentration (Ca) measured among five contrasting faba bean genotypes under rainfed and irrigated conditions at Narrabri, NSW.

**Genotypes**	**Irrigated**				**Rainfed**			
	**E (mmol m^**−2**^ ms^**−1**^)**	**A (μmol m^**−2**^ ms^**−1**^)**	**g_**s**_ (mmol m^**−2**^ ms^**−1**^)**	**Ci/ca**	**E (mmol m^**−2**^ ms^**−1**^)**	**A (μmol m^**−2**^ ms^**−1**^)**	**g_**s**_ (mmol m^**−2**^ ms^**−1**^)**	**Ci/ca**
11NF010c-4	1.47 ± 0.09 a	12.11 ± 1.10 bc	202.86 ± 37.93 a	0.69 ± 0.04 ab	0.88 ± 0.03 bcd	13.06 ± 1.01 abc	163.34 ± 7.45 abc	0.72 ± 0.04 a
11NF008b15	0.77 ± 0.11 cd	12.72 ± 1.09 bc	180.07 ± 22.61 abc	0.69 ± 0.03 ab	0.58 ± 0.17 d	8.94 ± 1.71 d	174.39 ± 25.41 abc	0.61 ± 0.05 b
AC0805#4912	0.97 ± 0.11 bcd	15.36 ± 1.55 a	161.98 ± 22.69 abc	0.74 ± 0.03 a	0.96 ± 0.19 bcd	11.22 ± 1.51 cd	152.07 ± 47.34 bcd	0.66 ± 0.05 ab
11NF020a-1	1.25 ± 0.22 ab	13.74 ± 1.42 ab	192.64 ± 30.32 ab	0.71 ± 0.02 a	1.03 ± 0.20 bc	9.11 ± 2.17 d	103.06 ± 20.46 d	0.74 ± 0.06 a
PBA Warda	1.00 ± 0.17 bcd	12.87 ± 2.64 abc	188.90 ± 52.02 ab	0.73 ± 0.04 a	0.99 ± 0.20 bcd	11.74 ± 1.46 bc	140.84 ± 21.12 cd	0.60 ± 0.05 b

Mean was calculated from the data recorded five times over the day (9:00–16:00). The measurement was done at the three-pod stage; plant age was between 120 and 128 days. Standard errors of the mean are shown.

Means followed by different letters within a column are significantly different at *p* < 0.05 by DMRT test.

The mean g_s_ among genotypes was higher in the irrigated trial compared to the rain-fed one. The highest g_s_ was observed in the genotype AC0805#4912, whereas the lowest was found in 11NF010c-4 across the day in both irrigated and rainfed trials. During midday, a sharp decline in g_s_ was observed for both trials (Supplementary Table 2).

The ratio of ci/ca translated the same observation as that of the net photosynthetic rate. The average ci/ca was slightly higher in the irrigated trial compared to the rainfed one. Among genotypes, AC0805#4912 had higher values for both trials while 11NF008b-15 had the lowest value for both trials. Statistically significant differences were observed among the genotypes for all tested gas exchange parameters (A, g_s_, and ci/ca). Genotype and treatment interactions were also found to be significant (Table 1).

### Concentrations of major sugars

**Figure 4:**
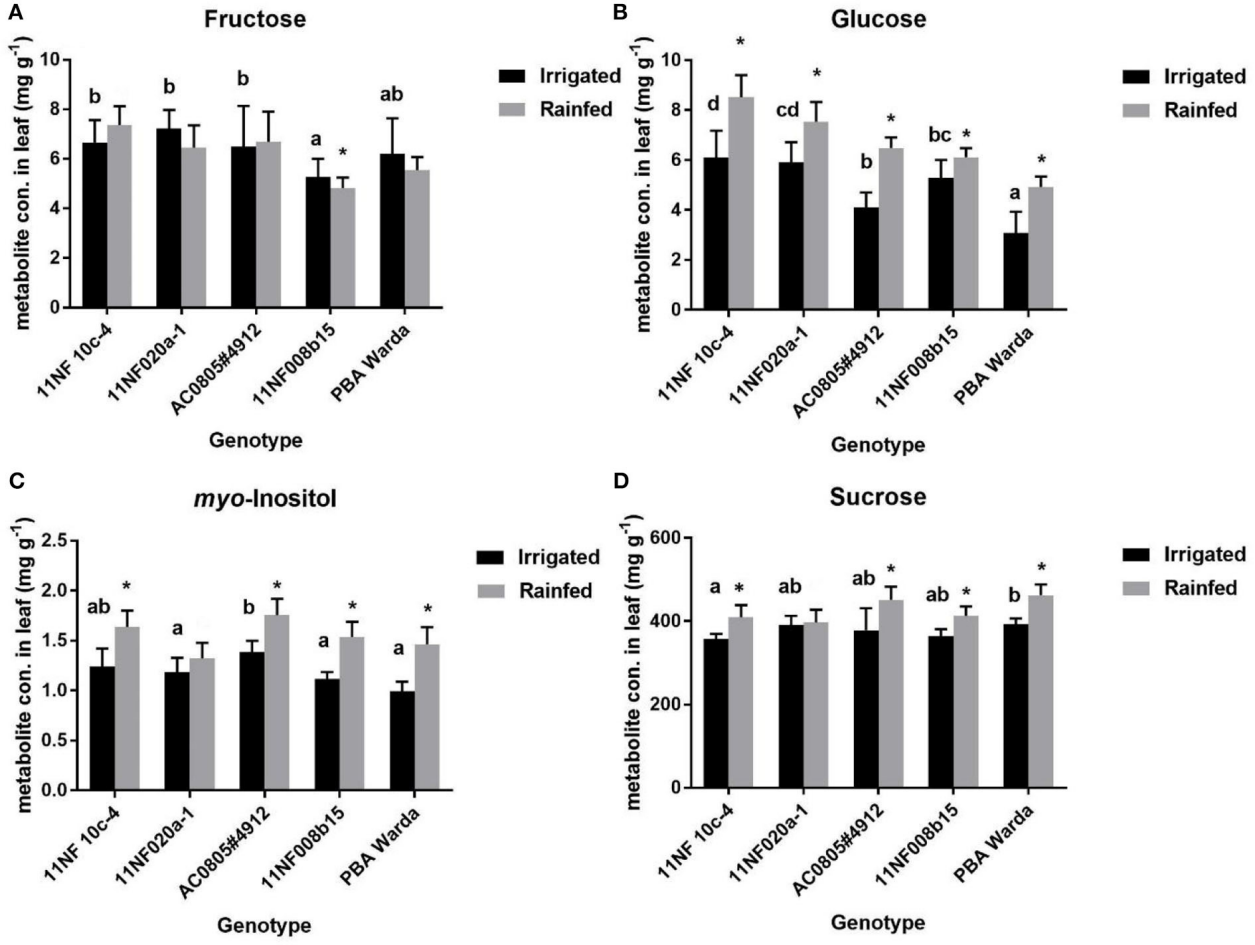
Average metabolite concentrations (mg g^−1^ leaf material) in contrasting faba bean genotypes under rainfed and irrigated conditions. Error bars were calculated from four replicates and represent standard error of the mean. Different letters denote a significant difference (*p* < 0.05) among genotypes calculated through DMRT. Asterisks (*) represent significance between irrigated and rainfed treatments. **(A)** Fructose, **(B)** Glucose, **(C)** myo-Inositol and **(D)** Sucrose.

Significant changes were found among treatments in most of the identified carbohydrates, except fructose, in both trials (Figure 4). Generally, soluble sugar concentrations were higher in the rainfed treatment compared to the irrigated treatment. The fructose concentration for the genotypes PBA Warda, 11NF008b-15, and 11NF020a-1 showed an opposite trend: higher in the irrigated trial compared to the rainfed trial. The genotype 11NF008b-15 had the lowest fructose concentration of all the other studied genotypes. The treatment effect was found to be significant for the genotypes 11NF008b-15 and 11NF010c-4 (Figure 4A). The concentration of glucose was found to be significant for both genotype and treatments. The highest concentration was found in the genotypes 11NF010c-4 and the lowest in PBA Warda (Figure 4B). *myo-*Inositol accumulation also followed that of glucose accumulation pattern; it was significantly higher in the rainfed trial than the irrigated trial. The genotype AC0805#4912 significantly differed from the other genotypes with the highest concentration among the studied genotypes (Figure 4C). Among the identified metabolites, sucrose was found to be the most abundant metabolite and was also observed in increased amounts in the rainfed trial for all genotypes. The WD-sensitive genotype 11NF010c-4 had significantly lower concentrations of sucrose compared to all the other studied genotypes with the highest concentration observed in PBA Warda (Figure 4D).

### Measured vs. predicted WUE

WUE measured using gas exchange and WUE calculated from the bulk leaf-level carbon isotope abundance were similar for both rainfed and irrigated trials. WUE calculation based on modeled assimilation weighted Ci is higher than that calculated from bulk leaf Δ. Both the predicted and measured WUE did not clearly define the treatment effects. All relationships except for PBA Warda were found to be significant (*p* < 0.05).

### Yield and biomass production under rainfed and irrigated conditions

**Figure 5:**
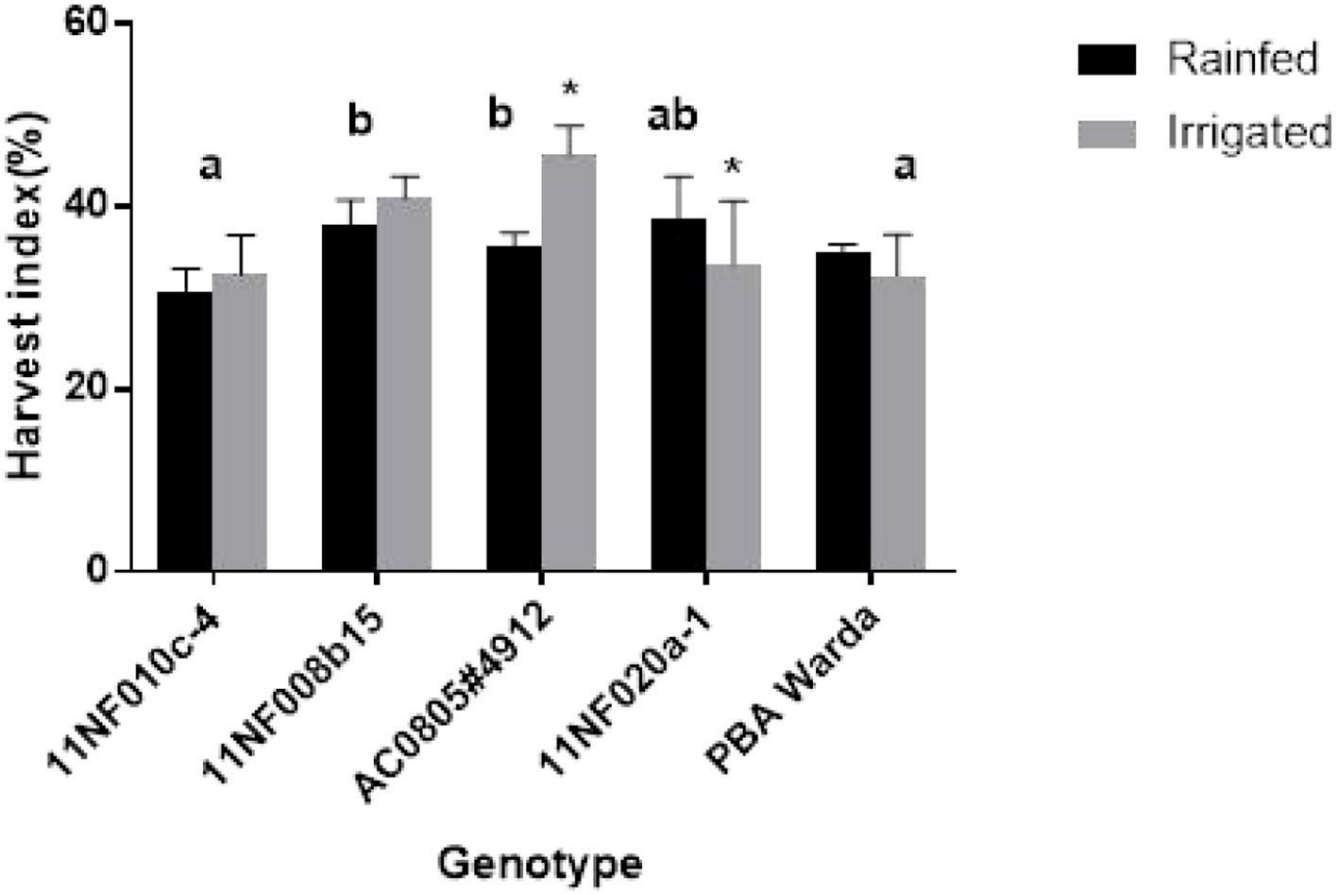
Harvest index (HI) (%) of contrasting faba bean genotypes grown under field condition (irrigated and rainfed trial) at Narrabri, Australia. Error bars were calculated from four replicates and represent standard error of the mean. Different letters represent a significant difference (*p* < 0.05) among genotypes calculated through DMRT. Asterisks (*) represent significance between irrigated and rainfed treatments.

Significant differences in HI were observed among genotypes. Treatments also elicited a significant influence on each of the genotypes AC0805#4912 and 11NF020a-1. The highest HI was found for the genotype AC0805#4912 and the lowest for 11NF010c-4 and PBA Warda (Figure 5). Generally, HI was higher in the irrigated trial except for genotypes 11NF020a-1 and PBA Warda.

**Figure 6:**
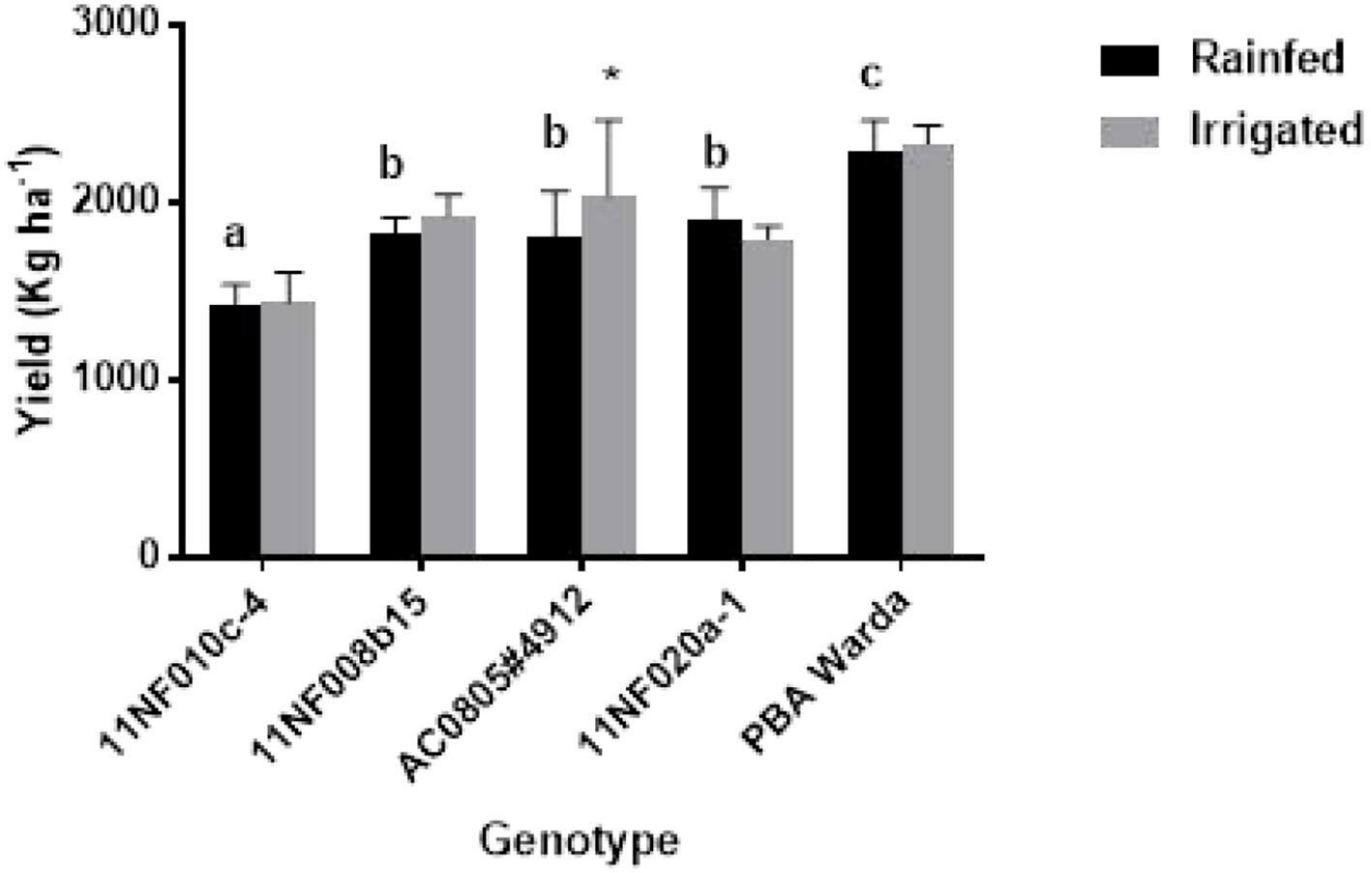
Yield (kg ha^−1^) of contrasting faba bean genotypes grown under field conditions (irrigated and rainfed trial) at Narrabri, Australia. Error bars were calculated from four replicates and represent standard error of the mean. Letters denote a significant difference (*p* < 0.05) among genotypes calculated through DMRT. Asterisks (*) represent significance between irrigated and rainfed treatments.

Genotypic variation for grain yield was significant among the tested genotypes. PBA Warda had the highest grain yield and 11NF010c-4 had the lowest. Irrigated plots produced higher yield compared to the rainfed plots, but were not statistically significant. Only genotype AC0805#4912 had significantly responded against treatment, producing more yields under irrigation. It was also observed that rainfed plots produced more yield for the genotype 11NF020a-1 though they were nonsignificant (Figure 6).

## Discussion

The accumulation of metabolites and changes in the amount of naturally occurring carbon isotopes represents two of the primary mechanisms by which plants may indicate the elicitation of physiochemical responses that enable acclimation to change in water availability. Quantification of metabolites among genotypes may be useful to detect biochemical markers as they are linked with phenotypic expression (Steinfath et al., 2010). In this study, we found that glucose, *myo*-inositol, and sucrose levels were significantly decreased under irrigated conditions compared to rainfed conditions. Furthermore, we identified that carbon isotope abundance among faba bean genotypes reflects a daily integral of WUE, with the incorporation of a relatively consistent offset to that measured by leaf-level gas exchange. Combined, these results illustrate the potential for biochemical and isotope analysis to determine novel pre-breeding traits to improve faba bean production under water-limited conditions.

### Physiological responses and biomass production among faba bean genotypes

One of the earliest plant defense mechanisms during WD is stomatal closure (Reynolds-Henne et al., 2010). The sensitivity of g_s_ to leaf water loss, particularly for 11NF020a-1, is an indication of this genotype being sensitive to WD. Stomatal response to WD generally involves stomatal closure to reduce dehydration. However, this closure also controls CO_2_ movement, ultimately influencing carboxylation and plant metabolism (Buckley, 2005). Complete stomatal closure was not observed in our rainfed trial throughout the day, indicating that this investigation was suitable for the study of plant physiological responses within the boundaries of physiological function. A sharp decline was observed in g_s_ in the midday, which is a commonly observed phenomenon for stomata due to the higher temperature as well as WD. Under field conditions, gas exchange is influenced by light intensity, heat, and/or relative humidity (Chaves et al., 2003). Instability of gas exchange measurements may occur due to physiological and short-term physiochemical changes, like alteration in water potential (Farooq et al., 2009) to alleviate the WD effect under field conditions. Such variation exhibited on times scales of minutes and influenced by diurnal patterns remains a major challenge to developing reliable tools to assess leaf-level gas exchange at the scale of a commercial breeding program.

Only genotype AC0805#4912 exhibited an increase in yield in response to the additional 25-mm water supplement (Figure 6) with rainfed plants producing comparable yield weights. Treatment responses to biomass production were not significant for genotypes, except AC0805#4912 and 11NF020a-1 (Figure 5), where the irrigated trial had a higher biomass compared to rainfed trial. Indeterminate growth of faba bean makes it sensible for the genotypes to produce higher biomass with the presence of adequate moisture. Moreover, under field conditions, water-holding capacity and high soil volume contribute to sustaining yield in the rainfed trial.

### Biochemical responses among faba bean genotypes

Identification and quantification of metabolites from field-grown samples provided meaningful insight into plant behavior in the cropping system. Leaf sample integrity may be compromised under field-based conditions due to sampling strategy or the interpretation of results in response to prevalent relative humidity, light, temperature, and soil nutrient in both the short and long term (Poorter and Nagel, 2000). In this study, leaf sampling was restricted to within 1 h of the diurnal cycle (16:00–17:00) to minimize the influence of diurnal changes as a systematic error in the sampling strategy.

Sugar alcohol accumulation has been found to be an important trait under environmental fluctuations (Loescher, 1987; Merchant and Richter, 2011), and the distribution of these compounds follows taxonomical patterns of plants (Bieleski and Briggs, 2005; Merchant et al., 2007) correlating with increasing aridity and WD. Despite considerable promise of sugar alcohols conferring stress tolerance, few studies have investigated their accumulation under field conditions (Streeter et al., 2001). In contrast, carbohydrates such as glucose and sucrose are less associated as “stress metabolites” due to their participation in primary metabolism requiring tight regulation, and are therefore less likely to exhibit accumulations in response to abiotic stress. In this study, glucose, *myo*-inositol, and sucrose increased significantly in irrigated treatments for all the studied genotypes despite only a slight increase in moisture level in the irrigated trial. The reasons behind the significant alteration of metabolites may be co-occurrence of stress as well as short-term response toward stress. Under field conditions, the co-occurrence of stress (drought heat) is frequent and is often misleading at the level of the individual. Plant responses against two co-occurring abiotic stresses are common and with physiological responses difficult to separate as the plant responds to the concomitant effects (e.g., see Poorter and Nagel, 2000; Mittler, 2006). Consequently, many studies of biochemical perturbations in response to stress conditions cannot be extrapolated to the field (e.g., Rizhsky et al., 2004). In this study, this suggests that, although our WD was relatively mild, the observed changes may be influenced by other environmental effects. For example, the accumulation of sugar alcohols and carbohydrates in this study may support what is observed among other sugar alcohol-accumulating species where accumulation is functioning as a supply of substrate for metabolism in sink tissues, thereby alleviating sugar-mediated suppression of photosynthesis under stress (Dumschott et al., 2018). Future studies should target both influence of individual stresses and plant developmental stages on metabolite accumulation in this crop and genetic variation governing these properties.

### Predictions of WUE based on the isotopic abundance

Carbon isotope abundance (δ^13^C) of leaf material to predict WUE is a well-established technique for a wide array of plant species (Seibt et al., 2008). More recently, the use of heterotrophic tissues to increase the precision of WUE calculations has been achieved in legume crops (Lockhart et al., 2016; Smith et al., 2016). Carbon isotope discrimination fluctuates with water availability primarily due to the effect of stomatal aperture influencing diffusional fractionation; therefore, it can be used to predict spatial and temporal carbon–water relationships (Seibt et al., 2008). Under the conditions experienced in this study, no treatment effect in gas exchange parameters was observed, likely due to the large variation in instantaneous measures of A/g_s_. The main advantage of Δ^13^C to assess WUE over gas exchange is that it assesses the properties of a carbon pool that is representative (hence integrative) across time (days to weeks), and therefore may reflect more meaningful changes in plant gas exchange than that of direct point-in-time measures by gas exchange.

**Figure 7:**
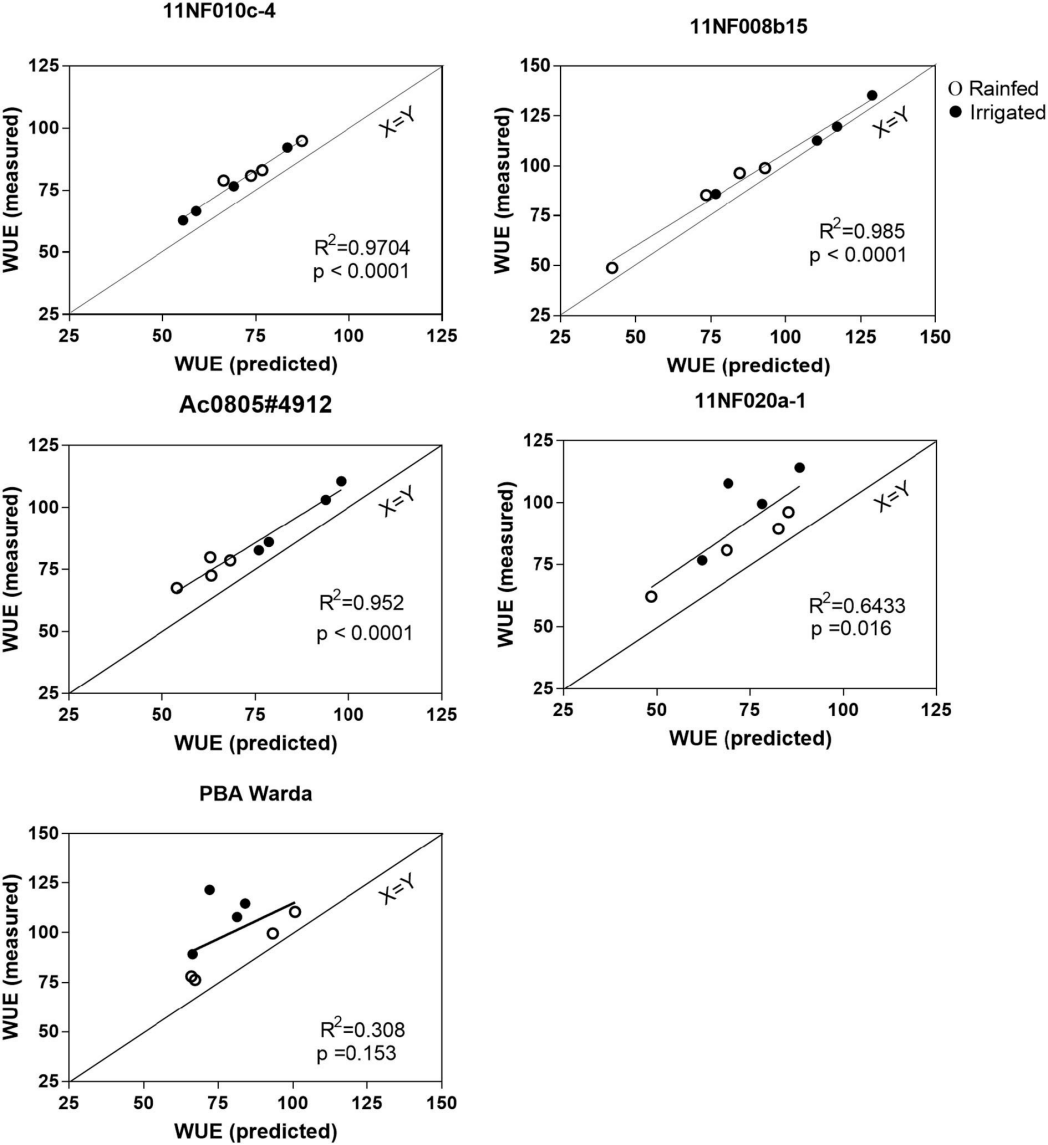
Predicted water use efficiency vs. measured water use efficiency has grown in irrigated and rainfed conditions of contrasting faba bean genotypes. Water use efficiency was calculated according to the formula of Farquhar and Richards (1984) and Seibt et al. (2008). The measurement was completed at the three-pod stage; plant age was between 120 and 128 days.

Due to frequent rainfall during the growing season, no significant treatment effects were observed in this study; however, an array of gas exchange rates was observed among the genotypes. Predicted WUE (from isotope abundance) was highly correlated with the measured (gas exchange) WUE values for all genotypes in this study, except PBA Warda (Figure 7). A consistent offset was observed between measured and predicted WUE. The offset between predicted and measured on the full carbon isotope model may be attributed to the parameterization of the model (such as the fractionation of isotopes attributable to phosphoenolpyruvate carboxylase) and the inclusion of fractionation events that are currently not contained within the model (e.g., see “parameter”; Farquhar and Sharkey, 1982). A number of established implications and indirect parameters counted within the model may be an issue while interpreting the outcome (Seibt et al., 2008). Incorporating post-photosynthetic fractionation in the model and parameterizing processes governing post-photosynthetic fractionation is a significant challenge (e.g., see Cernusak et al., 2009). Similarly, RuBisCO and PEP fractionation are hard to measure (Farquhar et al., 1989). In addition, parameterization of post-photosynthetic fractionation is likely to be species-specific due to the differences in biochemical fractionation during metabolite synthesis. Nevertheless, the relatively consistent offset observed here suggests that post-photosynthetic fractionation may be incorporated into models for the calculation of WUE with the inclusion of such caveats for screening applications such as those suggested here. An improved understanding in this area will help to develop cost-effective and reproducible tools to study plant carbon–water relationships.

## Conclusion

Leaf-level soluble chemistry of field-grown faba bean contains several indicators of plant performance and physiological health. Plant metabolite abundance, particularly that of low-molecular-weight carbohydrates and sugar alcohols, indicates physiochemical acclimation to the combined effects of drought (water, heat, light) and point-in-time measures of concentration are only indicative of higher synthesis rates. Consequently, increased concentration can only be correlative evidence of enhanced stress tolerance. δ^13^C is a reliable indicator of WUE. The consistent offset between predicted and measured WUE indicates that heterotrophic tissues may be used in the assessment of WUE across a large number of genotypes and is representative of temporal variation. When combined, this represents considerable use in screening for plant performance under field-grown conditions.

## Data availability statement

The original contributions presented in the study are included in the article/Supplementary material, further inquiries can be directed to the corresponding author/s.

## Author contributions

MM conceived the ideas, designed the research, conducted the experiments, analyzed the data, and wrote the first draft. KA and AM supervised the experiments and provided available resources. AS assisted in designing a part of the research and provided resources to conduct field experiments. AS, KA, and AM critically reviewed the manuscript. MT and LH did the data analysis, reconstructed the paper, and revised the paper. All authors contributed to the article and approved the submitted version.

## Conflict of interest

The authors declare that the research was conducted in the absence of any commercial or financial relationships that could be construed as a potential conflict of interest.

## Publisher's note

All claims expressed in this article are solely those of the authors and do not necessarily represent those of their affiliated organizations, or those of the publisher, the editors and the reviewers. Any product that may be evaluated in this article, or claim that may be made by its manufacturer, is not guaranteed or endorsed by the publisher.
