# Supplementary material for: Carbon isotope and soluble metabolites reflect physiological status among contrasting faba bean genotypes in response to water deficit
Source: Front Plant Sci. 2022 Sep 16;13:955406. doi: 10.3389/fpls.2022.955406 (PMC9523585; doi:10.3389/fpls.2022.955406)
Supplement: Supplementary file 1 [file Data_Sheet_1.docx]

Table 1: Mean leaf Δ^13^C (‰) of five contrasting faba bean genotypes under rainfed and irrigated conditions at Narrabri, NSW. Leaf sample was taken at the three-pod stage; plant age was between 120-128 days. Standard error is shown

| Genotypes | Irrigated | Rainfed |
| --- | --- | --- |
|  | Leaf Δ^13^C (‰) | Leaf Δ^13^C (‰) |
| 11NF010c-4 | -29.91±0.04 | -28.78±0.05 |
| 11NF008b15 | -28.18±0.03 | -28.78±0.05 |
| AC0805#4912 | -29.10±0.05 | -28.08±0.06 |
| 11NF020a-1 | -28.60±0.05 | -28.97±0.04 |
| PBA Warda | -28.49±0.02 | -28.22±0.05 |

Table 1a: Pedigree of the selected genotypes in field trial 2016

| Genotypes | Pedigree | Origin (country/region/state) |
| --- | --- | --- |
| AC0805#4912 | Seln ex ILB445 | Yemen |
| 11NF020a-1 | 453/4-2 X 331b/1-1 | Narrabri, NSW, Australia |
| 11NF010c-4 | 331b/1-1X220d/2-5 | Narrabri, NSW, Australia |
| 11NF008b-15 | AF07091X114/1-16 | Narrabri, NSW, Australia |
| PBA Warda | SP99046 × SP99081 | Narrabri, NSW, Australia |

**Table 2**: Mean transpiration rate (E, mmol m^-2^ ms^-1^), net photosynthetic rate (A, μmol m^-2^ ms^-1^), Stomatal conductance (g_s_, mmol m^-2^ ms^-1^) and ratio between intercellular carbon concentrations (Ci) to atmospheric carbon concentration (Ca) measured across the day five times among five contrasting faba bean genotypes under rainfed and irrigated conditions at Narrabri, NSW. Data recorded five times over the day (9.00 am to 4.00 pm). The measurement was done at the three-pod stage; plant age was between 120-128 days. Standard error are shown.

| Genotype | Time after sunrise (hours) | Irrigated | | | | Rainfed | | | |
| --- | --- | --- | --- | --- | --- | --- | --- | --- | --- |
|  |  | E (mmol m^-2^ ms^-1^) | A (μmol m^-2^ ms^-1^) | g_s_ (mmol m^-2^ ms^-1^) | Ci/Ca | E (mmol m^-2^ ms^-1^) | A (μmol m^-2^ ms^-1^) | g_s_ (mmol m^-2^ ms^-1^) | Ci/Ca |
| 11NF 10c-4 | 2 | 1.18 | 10.79 | 220.45 | 0.83 | 1.29 | 11.39 | 111.95 | 0.89 |
| 11NF 10c-4 | 4 | 0.92 | 11.95 | 156.31 | 0.71 | 0.88 | 9.26 | 140.54 | 0.68 |
| 11NF 10c-4 | 6 | 0.55 | 12.01 | 119.80 | 0.60 | 1.00 | 8.35 | 105.48 | 0.61 |
| 11NF 10c-4 | 8 | 1.07 | 14.36 | 160.37 | 0.62 | 0.62 | 8.08 | 92.20 | 0.73 |
| 11NF 10c-4 | 10 | 1.15 | 11.42 | 152.97 | 0.72 | 0.60 | 7.64 | 65.15 | 0.69 |
| 11NF008b15 | 2 | 0.76 | 12.56 | 212.55 | 0.81 | 1.09 | 9.38 | 184.37 | 0.76 |
| 11NF008b15 | 4 | 0.52 | 14.02 | 134.44 | 0.69 | 0.53 | 12.43 | 246.55 | 0.53 |
| 11NF008b15 | 6 | 0.54 | 11.28 | 120.83 | 0.62 | 0.84 | 13.88 | 181.11 | 0.68 |
| 11NF008b15 | 8 | 0.91 | 13.32 | 208.36 | 0.69 | 0.21 | 10.70 | 148.51 | 0.53 |
| 11NF008b15 | 10 | 1.13 | 12.42 | 224.17 | 0.66 | 0.22 | 9.70 | 111.44 | 0.56 |
| AC0805#4912 | 2 | 1.63 | 12.51 | 204.50 | 0.75 | 0.97 | 10.88 | 142.86 | 0.71 |
| AC0805#4912 | 4 | 1.54 | 15.26 | 291.83 | 0.85 | 1.06 | 15.97 | 174.47 | 0.55 |
| AC0805#4912 | 6 | 1.68 | 18.16 | 207.00 | 0.77 | 0.96 | 14.43 | 170.21 | 0.65 |
| AC0805#4912 | 8 | 1.17 | 16.26 | 168.56 | 0.64 | 0.94 | 12.51 | 168.48 | 0.71 |
| AC0805#4912 | 10 | 1.37 | 14.59 | 142.39 | 0.68 | 0.87 | 11.50 | 160.68 | 0.68 |
| 11NF020a-1 | 2 | 1.89 | 15.00 | 309.61 | 0.79 | 1.08 | 7.48 | 160.84 | 0.83 |
| 11NF020a-1 | 4 | 1.10 | 11.41 | 169.46 | 0.72 | 1.74 | 12.34 | 205.24 | 0.67 |
| 11NF020a-1 | 6 | 1.51 | 15.04 | 197.17 | 0.68 | 1.12 | 10.29 | 162.24 | 0.89 |
| 11NF020a-1 | 8 | 0.54 | 9.90 | 134.23 | 0.70 | 0.62 | 8.41 | 127.04 | 0.78 |
| 11NF020a-1 | 10 | 1.20 | 17.85 | 240.43 | 0.69 | 0.60 | 7.01 | 105.00 | 0.53 |
| PBA Warda | 2 | 2.11 | 12.65 | 220.56 | 0.73 | 1.45 | 7.22 | 161.93 | 0.69 |
| PBA Warda | 4 | 0.29 | 13.61 | 273.39 | 0.78 | 0.82 | 12.10 | 121.60 | 0.59 |
| PBA Warda | 6 | 0.78 | 15.92 | 178.63 | 0.73 | 1.43 | 16.39 | 213.72 | 0.65 |
| PBA Warda | 8 | 0.55 | 11.95 | 158.91 | 0.72 | 0.65 | 10.96 | 101.68 | 0.56 |
| PBA Warda | 10 | 1.27 | 10.23 | 144.66 | 0.68 | 0.60 | 12.01 | 105.25 | 0.53 |
